# Tibial acceleration variability during consecutive gait cycles is influenced by the menstrual cycle

**DOI:** 10.1186/1757-1146-4-S1-O3

**Published:** 2011-05-20

**Authors:** Simon Bartold, Adam Bryant, Ross Clark

**Affiliations:** 1 The University of Melbourne, Parkville, Victoria, 3010, Australia

## Background

The relationship between the phases of the menstrual cycle and injury risk remains unclear. Neuromuscular function may be compromised during menstruation, which could result in reduced cyclicality of movement patterns. We hypothesize that mediolateral (varus/valgus) knee acceleration during running gait will possess increased variability during menstruation when compared to ≈ovulation in women who do not take the monophasic oral contraceptive pill (MOCP).

## Methods

Thirty-six women (18 MOCP users: MOCP group and 18 non-pill users: NP group) performed six minute treadmill running trials at 10 km h−1 with an accelerometer fixed to the proximal tibia. Trials were performed at menstruation and ≈ovulation (for the MOCP group at a similar stage of the cycle) in a randomized order. The cyclicality of gross mediolateral tibial acceleration during 15 consecutive strides was assessed using combined wavelet and autocorrelation analysis. Longitudinal and anteroposterior accelerations were also examined. Repeated measures analysis of variance (ANOVA) tests were performed to assess differences at each stage of the menstrual cycle (α=0.05).

## Results

Gross mediolateral acceleration in the NP group had significantly (P=0.022) increased variability at the time of menstruation compared to ≈ovulation, and was also significantly (P=0.011) more variable than the MOCP group at the corresponding time point. No significant difference was observed for any measure in the MOCP group.

## Conclusions

Increased variability in the NP users at menstruation may be a result of compromised motor control strategies. This provides further evidence of variability in performance and motor control during menstruation, and may have implications for a female athlete's risk of injury, particularly at the level of the knee.

